# Dysregulated metabolic sensing of appetite in anorexia nervosa: implications of LEAP-2 regulation

**DOI:** 10.1192/j.eurpsy.2024.34

**Published:** 2024-08-27

**Authors:** C. Tezenas Du Montcel

**Affiliations:** ^1^Genetic and epigenetic vulnerabilities to addictive and psychiatric disorder, Inserm UMR 1266; ^2^Cliniques des Maladies mentales et de l’Encéphale, GHU Paris Psychiatrie et Neurosciences, Paris, France

## Abstract

**Abstract:**

Growing interests on the role of metabolic sensors in anorexia nervosa led to implicate metabolic sensing as consequences of anorectic sensing but also in the perpetuation of the disorder. Ghrelin is an orexigenic peptide secreted by the fundic cells of the stomach in situation of fasting and kown to initiate food intake through its activity on hypothalamic and motivation aspect of food intake. A body opf evidenc previously showed that patients suffering from anorexia nervosa display high plasma levels of ghrelin correlated with the nutritionnal status but his orexigenic signal do not seem to modify restrictive behavior. LEAP-2 (Liver Expressed Antimicrobial Peptide 2) is a recently discovered endogenous ghrelmin antagonist, increased during overnutrition and that decreases food intake in humans and animals.

We explored ghanges of ghrelin and LEAP-2 in a longitudinal cohort of 30 patients suffering from anorexia nervosa during a 4 months refeeding program. We show abnormal regulation of LEAP-2 in patients with higher levels in acute stages that decrease with refeeding. This abnormal regulation was associated with early relapse in patients. This abnormal regulation could counteract with the orexigenic signal of ghrelin in patients.

We discuss these results in light with recent evidence on the consequences of LEAP-2 increase of food intake and hedonic feeding relevant in understanding anorexia nervosa.

**Disclosure of Interest:**

None Declared

